# Tratamento de lesões condrais focais na articulação patelofemoral com membrana de colágeno: Resultados clínicos e funcionais em seguimento de dois anos

**DOI:** 10.1055/s-0045-1809520

**Published:** 2025-06-23

**Authors:** Pedro Debieux, José Ricardo Dantas Moura Costa, Wesley Araujo Weis, Diego da Costa Astur, Camila Cohen Kaleka, Moisés Cohen

**Affiliations:** 1 Departamento de Cirurgia Ortopédica, Hospital Israelita Albert Einstein, São Paulo, SP, Brasil; 2 Departamento de Cirurgia Ortopédica, Hospital Beneficência Portuguesa de São Paulo, São Paulo, SP, Brasil; 3 Departamento de Cirurgia Ortopédica, Instituto Cohen, São Paulo, SP, Brasil; 4 Centro de Traumatologia do Esporte, Departamento de Ortopedia e Traumatologia, Escola Paulista de Medicina, Universidade Federal de São Paulo, São Paulo, SP, Brasil; 5 Departamento de Ortopedia, Empresa Cuiabana de Saúde Pública, Cuiabá, MT, Brasil

**Keywords:** cartilagem articular, condrogênese, traumatismos do joelho, cartilage, articular, chondrogenesis, knee injuries

## Abstract

**Objetivo:**

Avaliar os desfechos clínicos e funcionais de pacientes submetidos a tratamento cirúrgico para reparo de lesão condral focal na patela e na tróclea, pela técnica de condrogênese induzida por matriz autóloga ( *autologous matrix-induced chondrogenesis* , AMIC, em inglês), após um mínimo de 2 anos de tratamento.

**Métodos:**

Ao todo, 24 pacientes (25 joelhos) com idade média de 39,6 ± 4,7 anos foram submetidos à técnica AMIC patelofemoral e foram avaliados em um seguimento médio de 3,64 ± 0,65 anos. Os fatores dos pacientes, a morfologia da lesão e as medidas de resultado relatadas pelos pacientes, incluindo os escores do International Knee Documentation Committee (IKDC), de Tegner, de Kujala, de Fulkerson, de Lysholm, e a Escala Visual Analógica (EVA), foram coletados.

**Resultados:**

O sexo masculino representou 76% da amostra. O tamanho médio do defeito das lesões condrais foi de 1,99 ± 0,36 cm ^2^ . Todos os defeitos foram classificados como de grau IV, de acordo com a classificação de Outerbridge. No seguimento final, os pacientes apresentaram os seguintes aumentos na pontuação média nos escores: Kujala – de 61,9 para 87,9; IKDC – de 51,3 para 83,6; Lysholm – de 64,0 para 88,4; Tegner – de 4,04 para 5,12; Fulkerson – de 60,2 para 89,3; e EVA – de 5,6 para 1,24. Todos os resultados apresentaram significância estatística ( *p* < 0,05).

**Conclusão:**

A AMIC é um método seguro, eficaz e viável para o tratamento de defeitos condrais sintomáticos, de espessura total da cartilagem femoropatelar, em casos adequadamente selecionados, e resultou em melhora clínica e funcional em todos os critérios analisados.

## Abstract (en)

**Objective:**

To evaluate the clinical and functional outcomes of patients undergoing surgical treatment to repair focal chondral lesions in the patella and trochlea using the autologous matrix-induced chondrogenesis (AMIC) technique after a minimum follow-up of 2 years.

**Methods:**

A total of 24 patients (25 knees) with a mean age of 39.6 ± 4.7 years underwent the patellofemoral AMIC technique and evaluation over a mean follow-up of 3.64 ± 0.65 years. We collected data on patient factors, lesion morphology, and patient-reported outcome measures, including the International Knee Documentation Committee (IKDC), Tegner, Kujala, Fulkerson, and Lysholm scores, as well as the Visual Analog Scale (VAS).

**Results:**

Male subjects accounted for 76% of the sample. The mean defect size of the chondral lesions was of 1.99 ± 0.36 cm ^2^ . All defects were of grade IV according to the Outerbridge classification. At the last follow-up, patients showed the following mean increases in the scores: Kujala – from 61.9 to 87.9; IKDC –from 51.3 to 83.6; Lysholm –from 64.0 to 88.4; Tegner –from 4.04 to 5.12; Fulkerson –from 60.2 to 89.3; and VAS – from 5.6 to 1.24. All results were statistically significant ( *p* < 0.05).

**Conclusion:**

The AMIC technique is a safe, effective, and feasible method to treat symptomatic full-thickness chondral defects of the patellofemoral cartilage in properly-selected cases, and it resulted in clinical and functional improvement in all criteria under analysis.

## Introdução

A cartilagem articular é um tecido altamente especializado, que tem como principal função permitir o movimento multiplanar da articulação sob diversas condições de carga aplicada. 1 Outra função importante da cartilagem hialina é a sua capacidade de transmissão de carga ao osso subcondral adjacente sem causar danos. Além disso, ela é capaz de fornecer uma superfície lisa e lubrificada, de baixo atrito, que permite a manutenção da homeostase articular. 2

As lesões que acometem a região patelofemoral são encontradas em até um terço dos casos, e podem ser responsáveis por sintomas extremamente limitantes para os pacientes. 3 4 As causas mais comuns são trauma, luxação da patela, mau alinhamento e instabilidade, 3 5 e essas lesões ainda são um grande desafio para o cirurgião ortopédico. 6 7 8 Diversas formas de tratamento têm sido propostas, sendo a terapia cirúrgica recomendada nos casos em que as modalidades conservadoras não obtiveram sucesso. Entre as opções disponíveis, destacam-se as microperfurações, o implante autólogo de condrócitos e o enxerto osteocondral autólogo, e o tratamento ideal ainda é controverso. 3 9

A condrogênese induzida por matriz autóloga ( *autologous matrix-induced chondrogenesis* , AMIC, em inglês) entra nesse arsenal de técnicas que objetivam reparar lesões focais na espessura total da cartilagem. Essa técnica é realizada por meio da estimulação da medula óssea pelas microfraturas, juntamente com a cobertura da lesão com uma membrana de colágeno tipos I e III, que tem como objetivo conter o coágulo formado, o que promove aderência, proliferação e diferenciação celular. 7 8 9 10 11 12 13 Notadamente eficaz no tratamento das lesões condrais condilares, ainda existe uma lacuna de resultados para esta técnica quando se analisam isoladamente as lesões no compartimento patelofemoral. 9 14 Desde a sua introdução, contudo, a AMIC tem demonstrado bons resultados tanto no curto quanto no médio prazos. 7 8 10 15 16 17

Portanto, o objetivo deste estudo foi analisar os desfechos clínicos de pacientes submetidos a tratamento cirúrgico para reparo de lesão condral focal na patela e na tróclea, pela AMIC, após 2 anos do tratamento.

## Materiais e Métodos

Trata-se de uma análise descritiva e observacional do tipo série de casos. Os dados foram obtidos por meio da análise de prontuários de pacientes submetidos a tratamento cirúrgico para reparo de lesão condral focal na patela ou na tróclea, pela AMIC), de 2015 a 2020. O estudo foi aprovado pelo Comitê de Ética em Pesquisa institucional sob o número CAAE: 65062522.9.0000.5505.

Os 24 pacientes (25 joelhos) incluídos, 19 homens e 5 mulheres, foram operados por um único cirurgião e acompanhados por pelo menos 2 anos após o procedimento cirúrgico. Todos receberam orientação detalhada sobre a técnica cirúrgica proposta, bem como sobre outras opções de tratamento disponíveis, com suas respectivas vantagens e desvantagens, e consentiram com o procedimento escolhido.

Os critérios de inclusão abrangeram pacientes com idades entre 15 e 60 anos, diagnosticados com lesão condral focal na patela ou na tróclea, com área entre 0,5 cm ^2^ e 4,0 cm ^2^ , de graus III ou IV na classificação da International Cartilage Repair Society (ICRS, Sociedade Internacional de Reparo de Cartilagem), que mantinham atividade física regular e não responderam ao tratamento conservador com fisioterapia e reabilitação funcional por até 6 semanas. Foram excluídos pacientes com mau alinhamento patelar, lesões ósseas subcondrais (osteocondrais), osteoartrite avançada (grau ≥ 2 na classificação radiográfica de Ahlbäck), histórico de reparos cartilaginosos ou de procedimentos concomitantes, como reparo meniscal ou reconstrução ligamentar.

Todos os pacientes com suspeita de lesão condral na patela ou na tróclea foram submetidos a avaliação pré-operatória, que incluiu radiografias e exames de ressonância magnética (RM), para a caracterização e a medição da lesão condral, além da identificação de possíveis lesões ligamentares ou mau alinhamento nos membros inferiores. Essa abordagem permitiu a seleção criteriosa dos participantes que preenchiam os critérios de inclusão.

## Técnica Cirúrgica

**Fig. 1:**
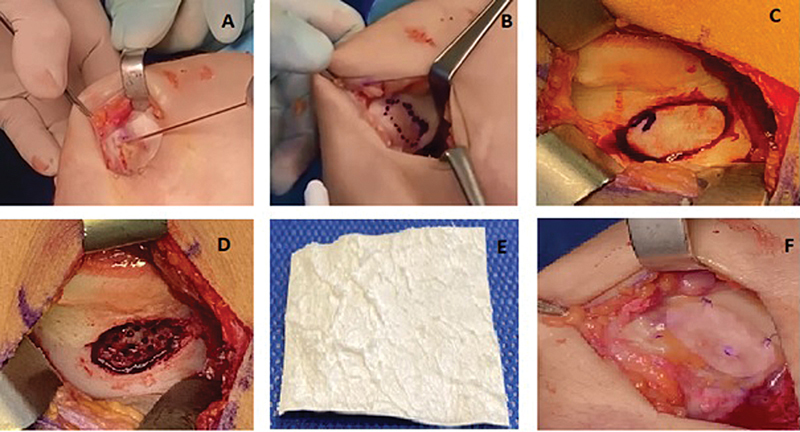
Imagem clínica de AMIC em lesão de cartilagem. **A** , extenso defeito na cartilagem. **B** , regularização de bordas da lesão. **C** , marcação de tamanho da lesão após curetagem. **D** , nanoperfuração do osso subcondral. **E** , membrana de colágeno porcino tipo I/III. **F** , aspecto final após implante de membrana associada à sutura monofilamentar 5-0, reforçada com cola de fibrina.

Inicia-se com uma artroscopia para a avaliação detalhada da lesão condral e a confirmação diagnóstica, seguida de artrotomia parapatelar medial ou lateral, conforme a localização da lesão. Após eversão da patela, as bordas da lesão são moldadas para criar paredes verticais estáveis de cartilagem adjacente saudável ( Fig. 1 ). O defeito é marcado e curetado até a sua camada calcificada, e depois procede-se à nanoperfuração do osso subcondral. Em seguida, a membrana de colágeno porcino de tipos I e III (Chondro-Gide, Geistlich Pharma AG) é aplicada sobre a lesão e fixada com fio de sutura monofilamento 5–0 (PDS, Ethicon, Inc.) e reforçada com cola de fibrina (Tisseel, Baxter Medical Pharmaceutical Ltd.) nas bordas ( Fig. 1 ).

## Reabilitação

A reabilitação pós-operatória inclui imobilização inicial com órtese coxomaleolar por 10 dias, com liberação de carga total no membro operado. O ganho progressivo de amplitude de movimento é iniciado conforme a tolerância à dor, com deambulação sem órtese incentivada até a normalização da marcha entre 6 e 8 semanas. Os pacientes são liberados para esportes de contato de 6 a 8 meses após a cirurgia.

## Coleta de Dados

Dados demográficos como sexo, idade, lateralidade, localização da lesão, tamanho, tempo de acompanhamento e procedimentos associados foram coletados. A avaliação clínica pré-operatória foi feita por meio de escores clínicos padronizados internacionalmente, entre eles, os do International Knee Document Committee (IKDC), de Lysholm, de Fulkerson, de Kujala, de Tegner, e a Escala Visual Analógica (EVA). Os mesmos escores foram aplicados mais uma vez após 2 anos de seguimento ambulatorial.

## Análise Estatística

Para a análise estatística, foram usados os programas IBM SPSS Statistics for Windows, versão 20.0 (IBM Corp.), Minitab 16 (Minitab, LLC) e Excel 2010 (Microsoft Corp.). A análise das variáveis quantitativas foi feita mediante o cálculo de médias, desvios padrão (DPs) e quartis. Para as variáveis qualitativas, calcularam-se as frequências absolutas e relativas. Na comparação dos resultados entre os grupos, foram realizados os testes de Wilcoxon, de Mann-Whitney, e o de igualdade de duas proporções. Os dados foram demonstrados em tabelas e diagramas de caixa. O nível de significância estatística foi de 95%, e o valor de *p* foi definido como 0,05.

## Resultados

Foram analisados 24 pacientes (25 joelhos), com idades entre 15 e 60 (média: 39,6 ± 4,7) anos, sendo 5 mulheres e 19 homens. As lesões acometeram a patela em 12 casos e a tróclea femoral em 13 casos, com tamanho entre 1,0 cm ^2^ e 3,99 cm ^2^ na patela, e 0,9 cm ^2^ e 2,94 cm ^2^ na tróclea. O tempo médio de seguimento foi de 3,54 ± 0,65 anos. A Tabela 1 apresenta as frequências do local da lesão, da lateralidade e do sexo.

**Tabela 1:** Comparação entre os grupos quanto à distribuição dos fatores qualitativos

		Patela		Tróclea		Total	
		N	%	N	%	N	%
Lateralidade	Direita	7	58,3%	5	38,5%	12	48,0%
	Esquerda	5	41,7%	8	61,5%	13	52,0%
Sexo	Feminino	5	41,7%	1	7,7%	6	24,0%
	Masculino	7	58,3%	12	92,3%	19	76,0%
Local	Lateral	5	41,7%	5	38,5%	10	40,0%
	Medial	4	33,3%	3	23,1%	7	28,0%
	Medial e lateral	3	25,0%	5	38,5%	8	32,0%

Os escores do IKDC, de Lysholm, de Fulkerson, de Kujala, de Tegner, e a EVA foram avaliados no pré e no pós-operatórios, com um tempo mínimo de 24 meses. Houve aumento estatisticamente significativo em praticamente todas as comparações, sendo a única exceção o escore de Tegner para o grupo tróclea, em que não foi apresentada significância estatística, mesmo com o valor da média tendo aumentado de 4,46 para 5,31 ( *p* = 0,305). A Tabela 2 apresenta a comparação entre as médias para os grupos patela e tróclea.

**Tabela 2:** Comparação entre os escores por grupo

			Média	Mediana	Desvio padrão	N	*p*
Lysholm	Patela	Pré	60,1	62,5	± 13,6	12	0,003
		Pós	88,3	90,5	± 10,7	12	
	Tróclea	Pré	67,5	63,0	± 13,2	13	0,007
		Pós	88,5	89,0	± 13,4	13	
IKDC	Patela	Pré	48,7	46,6	± 14,9	12	0,002
		Pós	82,6	89,1	± 13,9	12	
	Tróclea	Pré	53,6	52,9	± 11,5	13	0,003
		Pós	84,6	92,0	± 14,5	13	
Tegner	Patela	Pré	3,58	3,00	± 1,62	12	0,017
		Pós	4,92	5,50	± 1,31	12	
	Tróclea	Pré	4,46	5,00	± 1,76	13	0,305
		Pós	5,31	5,00	± 1,93	13	
Fulkerson	Patela	Pré	56,9	60,5	± 15,0	12	0,002
		Pós	88,2	90,0	± 11,6	12	
	Tróclea	Pré	63,3	64,0	± 15,1	13	0,005
		Pós	90,3	94,0	± 14,4	13	
EVA	Patela	Pré	5,83	6,50	± 2,48	12	0,005
		Pós	1,42	0,50	± 1,93	12	
	Tróclea	Pré	5,38	6,00	± 2,06	13	0,003
		Pós	1,08	0,00	± 1,71	13	
Kujala	Patela	Pré	58,3	58,5	± 14,5	12	0,002
		Pós	86,4	89,0	± 12,9	12	
	Tróclea	Pré	65,2	70,0	± 14,1	13	0,006
		Pós	89,2	91,0	± 12,8	13	

**Abreviaturas:** EVA, Escala Visual Analógica; IKDC, International Knee Documentation Committee; Pós, pós-operatório; Pré, pré-operatório.

**Fig. 2:**
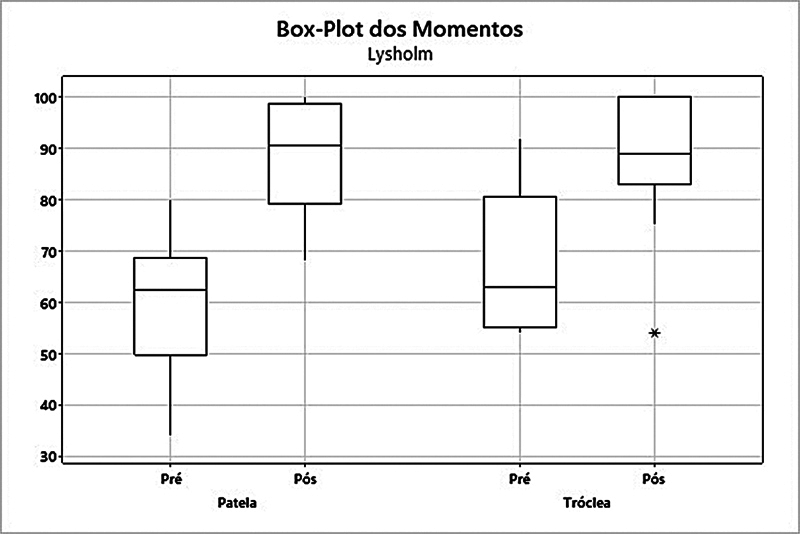
Análise dos grupos patela e tróclea quanto ao escore de Lysholm.

**Fig. 3:**
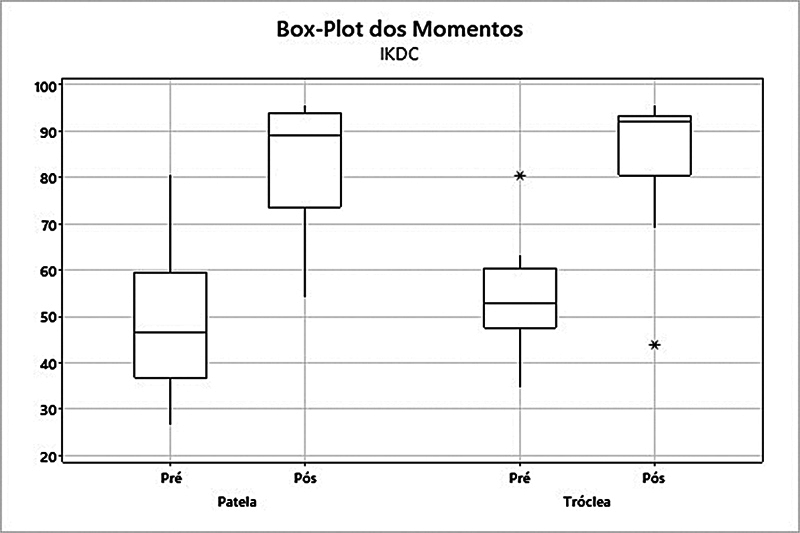
Análise dos grupos patela e tróclea quanto ao escore do International Knee Documentation Committee (IKDC).

**Fig. 4:**
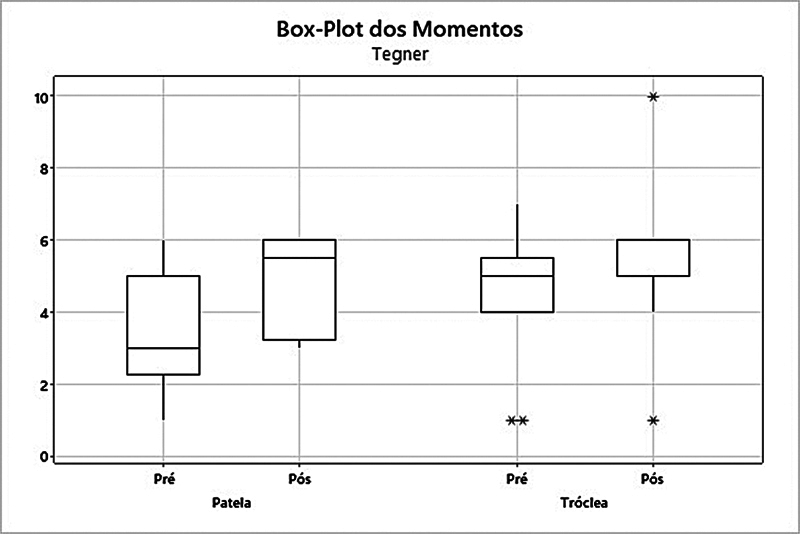
Análise dos grupos patela e tróclea quanto ao escore de Tegner.

Quando analisado o grupo patela, o escore de Lysholm apresentou um aumento da média de 60,1 para 88,3 ( *p* = 0,003). Já no grupo tróclea, a média subiu de 67,5 para 88,5 ( *p* = 0,007) ( Fig. 2 ). No escore IKDC no grupo patela, ocorreu um aumento da média de 48,7 para 82,6 ( *p* = 0,002). Já no grupo tróclea, a média subiu de 53,6 para 84,6 ( *p* = 0,003) ( Fig. 3 ). No escore de Tegner no grupo patela, ocorreu um aumento da média de 3,58 para 4,92 ( *p* = 0,017) ( Fig. 4 ).

**Fig. 5:**
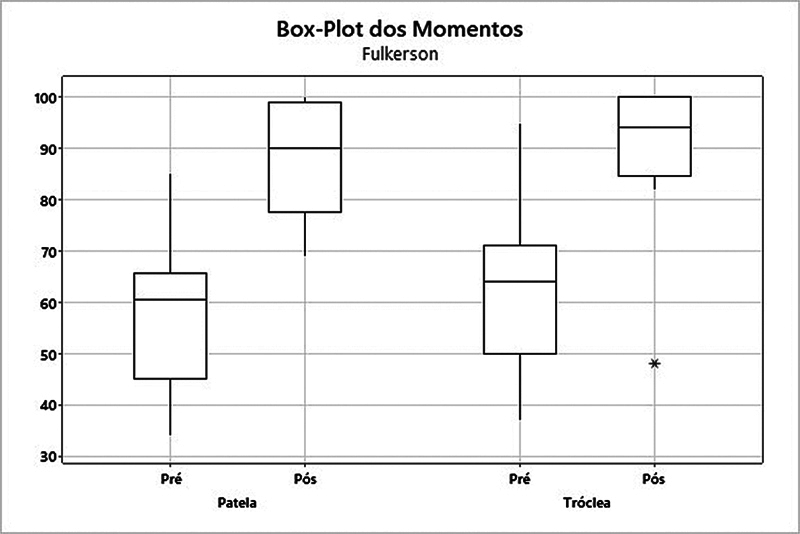
Análise dos grupos patela e tróclea quanto ao escore de Fulkerson.

**Fig. 6:**
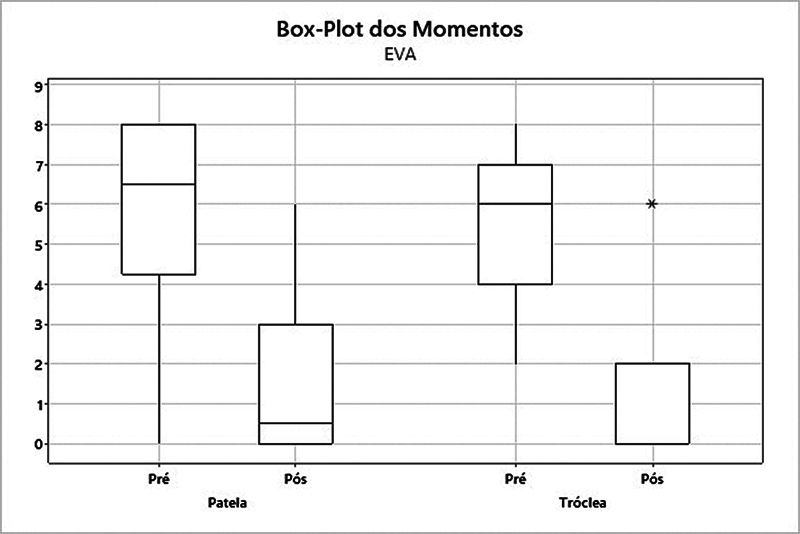
Análise dos grupos patela e tróclea quanto ao escore na Escala Visual Analógica (EVA).

**Fig. 7:**
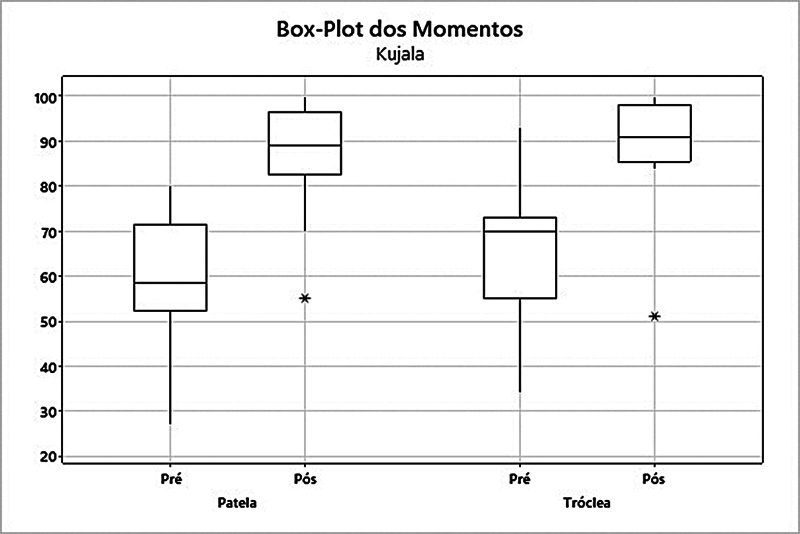
Análise dos grupos patela e tróclea quanto ao escore de Kujala.

Ao analisarmos o escore de Fulkerson, o grupo patela apresentou um aumento da média de 56,9 para 88,2 ( *p* = 0,002). Já no grupo tróclea, a média subiu de 63,3 para 90,3 ( *p* = 0,005) ( Fig. 5 ). Na EVA no grupo patela, ocorreu uma redução da média de 5,83 para 1,42 ( *p* = 0,005). Já no grupo tróclea, a média subiu de 5,38 para 1,08 ( *p* = 0,003) ( Fig. 6 ). Por fim, no escore de Kujala no grupo patela, houve um aumento da média de 58,3 para 86,4 ( *p* = 0,002). Já no grupo tróclea, a média subiu de 65,2 para 89,2 ( *p* = 0,006) ( Fig. 7 ).

## Discussão

O dado mais relevante encontrado neste estudo foi o de que a técnica AMIC foi eficaz no tratamento de lesões condrais de espessura total, na patela e tróclea, com seguimento mínimo de 2 anos. Em todos os escores analisados, ocorreram melhoras clínica e funcional estatisticamente significativas, o que resultou em redução dos sintomas e retorno ao esporte em níveis pré-lesão.

Corroborando esses achados, Gille et al. 18 conduziram um estudo com uma amostra de pacientes com lesões patelofemorais inferior à aqui obtida ( *n* = 11, contra *n* = 25 neste estudo), porém com idade média semelhante. Naquele, contudo, a patela foi predominantemente acometida (9 contra 2), enquanto neste, as lesões na tróclea foram mais frequentes (13 contra 12). Semelhantemente, Gille et al. 18 encontraram melhoras nos escores de Lysholm, com um aumento na média de 36 ± 21 no pré-operatório para 76 ± 24 no seguimento de 24 meses. Os escores de Tegner e do IKDC também apresentaram um aumento significativo para defeitos localizados na patela durante o mesmo período de análise, sendo esses dados publicados em artigo anterior. 10 Waltenspül et al., 3 analogamente, observaram aumento na média no escore de Kujala de 63,5 ± 11,6 no pré-operatório para 72,2 ± 17,4 após um acompanhamento mínimo de 24 meses ( *p* = 0,029).

No tocante à satisfação do paciente pós-AMIC, Panni et al., 19 em um seguimento médio de 7 anos, relataram que 76,2% dos pacientes consideraram o tratamento bom ou excelente. De forma similar, Gille et al., 18 ao avaliarem 27 pacientes tratados com AMIC, constataram que 87% estavam satisfeitos com os resultados. A análise deste estudo confirma esses dados, pois revelou resultados positivos, com 84% dos pacientes classificando sua satisfação como boa ou excelente, em consonância com a literatura supramencionada.

Em seguimento de 12 meses, Tradati et al. 20 relataram resultados significativos em pacientes submetidos à AMIC. O escore de Kujala passou de 49,6 no pré-operatório para 87,8 após 12 meses ( *p* < 0,01). O escore do IKDC aumentou de 36,1 para 79,8 ( *p* < 0,01). Além disso, houve redução significativa da dor, com o escore na EVA reduzindo de 7,5 para 1,5 ( *p* < 0,01). 20 Este estudo revela que os benefícios do tratamento proposto por Tradati et al. 20 se mantêm por pelo menos 24 meses, uma vez que as variações dos escores aqui aferidos são simétricas às observadas por aqueles autores.

O compartimento patelofemoral consiste na região do joelho mais desafiadora para o tratamento de lesões condrais. Hinckel et al. 21 conduziram uma revisão sistemática para decifrar qual a técnica mais adequada neste cenário. Observaram que o uso da membrana de colágeno associado ao implante autólogo de condrócitos ( *autologous chondrocyte implant* , ACI, em inglês) rendia resultados superiores aos das demais técnicas. A técnica AMIC, contudo, não foi incluída nessa análise 21 pela ausência de estudos controlados até então. De fato, o uso da AMIC está consagrado para a região condilar; Astur et al., 6 por exemplo, analisaram desfechos semelhantes aos relatados aqui, em 15 pacientes, por 12 meses, e notaram uma diferença de 4 pontos na pontuação na EVA, ao final do seguimento de 12 meses, análogo ao aqui encontrado após 24 meses, mesmo que aqui se trate do compartimento anterior do joelho.

A associação da AMIC clássica, aqui descrita, com os ortobiológicos, preservando ou não o osso subcondral, tem sido uma tendência. A técnica AMIC *plus* , por exemplo, associa o concentrado de aspirado de medula óssea ( *bone marrow aspirate concentrate* , BMAC, em inglês) à membrana de colágeno. Essa combinação tem apresentado evidências de alívio da dor pós-operatória e melhora funcional, além da manutenção dos resultados por até 3 anos. 22 Sciarretta et al. 23 relataram resultados promissores com o uso da técnica LIPO-AMIC, que combina a AMIC ao uso simultâneo de enxerto de tecido adiposo autólogo. Os autores 23 observaram melhora precoce e progressiva, além de alívio duradouro dos sintomas; além disso, notaram recuperação importante e a manutenção das atividades diárias funcionais e esportivas aos 2 anos e, especialmente, em acompanhamentos de 5 anos. Outra técnica que merece atenção é a do implante de cartilagem picada ( *minced cartilage implantation* , em inglês), que pode ser aplicada em defeitos de cartilagem de pequeno diâmetro. Essa técnica pode ser considerada uma alternativa aos procedimentos de estimulação da medula óssea, uma vez que o implante também pode ser realizado em procedimento de etapa única, por meio de técnicas até mesmo artroscópicas. Essa abordagem minimamente invasiva é de fácil aplicação e uma opção atraente para o tratamento de lesões condrais focais. 24

Este estudo não está isento de limitações, muitas delas inerentes ao seu próprio desenho, por se tratar de um estudo de série de casos do tipo observacional. Primeiramente, o número de participantes ( *n* = 25) pode limitar a robustez estatística, a despeito de ser um número relevante quando comparado aos da literatura internacional. Além disso, o fato de se tratar de um estudo de braço único sem um grupo de controle impede a comparação direta e a avaliação da eficácia relativa das intervenções. Uma limitação do estudo está na impossibilidade de comparação entre diferentes técnicas, como a nanofratura, uma vez que, para a análise de dados, os grupos de controle se tornam de suma importância. A heterogeneidade das lesões entre os pacientes constitui uma limitação significativa do estudo, uma vez que foram incluídas lesões localizadas tanto na tróclea quanto na patela. Adicionalmente, não foram consideradas variações discretas nos padrões anatômicos e biomecânicos da região patelofemoral. Outra limitação importante é a ausência de avaliação de desfechos por meio de imagem. Além disso, um seguimento mais prolongado poderia fornecer dados mais robustos e enriquecer as conclusões.

## Conclusão

O reparo de lesões condrais com a utilização de membrana de colágeno é uma técnica segura, eficaz e viável para o tratamento de defeitos condrais sintomáticos, da espessura total da cartilagem femoropatelar, em casos adequadamente selecionados, que resulta em melhoras clínica e funcional em todos os critérios analisados, após 2 anos de seguimento.
